# Case Report: Successful Management of Refractory Keratomycosis in an Alpaca Using Penetrating Keratoplasty and Combination Antifungal Therapy (Caspofungin 0.5% and Terbinafine 1%)

**DOI:** 10.3389/fvets.2021.644074

**Published:** 2021-03-11

**Authors:** Braidee C. Foote, Joe S. Smith, Anna Catherine Bowden, Rachel A. Allbaugh, Lionel Sebbag

**Affiliations:** ^1^Department of Veterinary Clinical Sciences, College of Veterinary Medicine, Iowa State University, Ames, IA, United States; ^2^Department of Biomedical Sciences, College of Veterinary Medicine, Iowa State University, Ames, IA, United States; ^3^Koret School of Veterinary Medicine, The Hebrew University of Jerusalem, Rehovot, Israel

**Keywords:** camelid ophthalmology, *Scopulariopsis brevicaulis*, *Fusarium verticillioides*, corneal abscess, corneal crosslinking, keratomalacia, fungal keratitis

## Abstract

Fungal keratitis is a common disease in certain parts of the world and affects several species, including equids, camelids, and homo sapiens, leading to blindness or loss of the eye if the infection is not adequately controlled. Reports of clinical use of antifungals caspofungin and terbinafine are limited across both veterinary and human medical literature. The alpaca presented in this case demonstrates that deep keratomycosis can be caused by *Scopulariopsis brevicaulis* and *Fusarium verticillioides*, two previously unreported fungi to cause keratomycosis in camelids. This report demonstrates successful management with a combination of surgery and topical ophthalmic treatment with caspofungin 0.5% solution and terbinafine 1% dermatologic cream, after initially failing treatment with topical voriconazole 1% solution. Combination therapy appears more effective than monotherapy with some fungal organisms, and synergy between antifungal agents is thought to play a role in the success of combination therapy. Surgery to remove the bulk of the fungal infection is especially helpful in cases that fail initial medical therapy.

## Background

New world camelids, especially alpacas, are increasingly popular as companion animals or for fiber production in North America (1). Like horses, alpacas have prominent eyes prone to traumatic corneal disease and risk secondary microbial infection (1, 2). Fungi are normal inhabitants of the ocular surface microflora in healthy New World camelids (56% positive growth). One study from the Northeastern United States described a 6.5% prevalence of fungal keratitis (keratomycosis) in camelids, similar to equine data (5.7–8.6% prevalence) (1, 3).

Keratomycosis occurs in humans also; however, the prevalence of fungal keratitis varies widely, due to climate, age, gender, socioeconomics, and urbanization (4–6). Keratomycosis can be challenging to manage and may result in a loss of vision or the eye if not treated appropriately. This report describes the successful management of an alpaca with a full-thickness corneal abscess associated with two fungi, *Scopulariopsis brevicaulis* and *Fusarium verticillioides*.

## Case Presentation

An 11-year-old female alpaca was presented to the Lloyd Veterinary Medical Center at the Iowa State University (LVMC-ISU) for evaluation of suspected corneal infection and recent weight loss. Ten days prior to referral, the owner noted that the alpaca had acute epiphora and blepharospasm of the right eye (OD). Topical neomycin-polymyxin B-bacitracin (NPB) ophthalmic ointment was initiated twice daily by the family veterinarian (rDVM) for a corneal ulcer OD diagnosed via fluorescein staining. Trauma was not observed but was the suspected cause given the species and group housing environment. Acute worsening of the ulcer occurred within 1 week and treatment was switched to topical ofloxacin 0.3% and atropine 1% (each twice daily) and oral meloxicam (60 mg once daily) until referral. No other diagnostics or treatments were performed by the rDVM.

**Figure 1:**
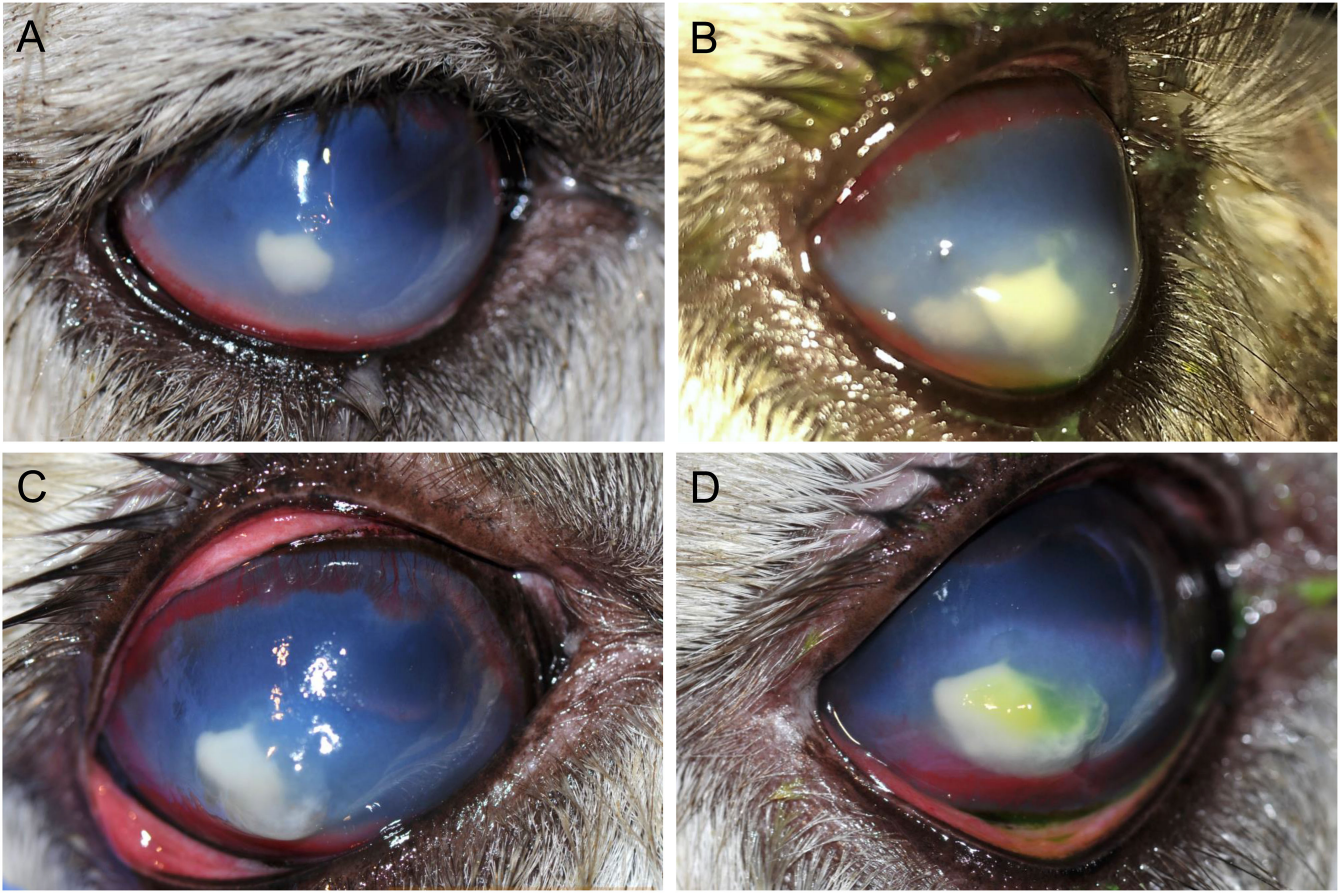
Photographs of the progression of a refractory fungal stromal abscess of the right eye in an 11-year-old female alpaca on day 1 **(A)**, day 2 **(B)**, day 3 **(C)**, and day 7 **(D)**. **(A)**–Day 1, on presentation, there is approximately a 5 mm paraxial stromal abscess with a yellow creamy appearance and fluffy borders, pinpoint satellite lesions in the adjacent stroma (unable to appreciate in photo), marked geographical corneal edema, and dense stromal corneal vascularization. Photo obtained prior to application of fluorescein stain. **(B)**–By day 2, a satellite lesion adjacent to the stromal abscess had substantially increased in size by roughly 3 mm and the multiple small corneal bullae had worsened/coalesced to become a large corneal bulla overlying the medial aspect of the lesion with fluorescein stain uptake. **(C)**–By day 3, despite aggressive medical management the two stromal abscesses had coalesced, the corneal bullae had progressed, and the overlying cornea appeared moderately malacic. Photo obtained prior to use of fluorescein stain; after application it was noted the ulcer had increased in size to cover the whole lesion. Corneal cross linking with the accelerated protocol was performed the following day. **(D)**–On day 7, the stromal abscess had continued to increase by ~2 mm with improvement in corneal bullae, malacia, and progression of stromal vascularization. Fluorescein staining is evident overlying a portion of the abscess.

On presentation (day 1), physical examination revealed a slightly low body condition score of 3/9; however, vitals, capillary refill time, mucus membrane color, FAMACHA score, cardiothoracic auscultation, and first compartment contractions were all within normal limits. Fecal parasitology assessment was previously performed by the rDVM. Ophthalmic examination was performed with slit lamp biomicroscopy (SL-17, Kowa, Tokyo, Japan) and indirect ophthalmoscopy (Keeler Vantage, Keeler instruments Inc., Broomall, PA, USA). The left eye (OS) was normal. OD had a positive but inconsistent menace response, positive palpebral and dazzle reflexes, and reduced direct and consensual (right to left) pupillary light reflexes. Moderate blepharospasm and mucopurulent discharge were present. The conjunctiva was moderately hyperemic, the cornea had a 5 × 6 mm yellow creamy paraxial stromal abscess with fluffy borders, a few surrounding pinpoint satellite lesions in the adjacent stroma, severe corneal edema with secondary bullae, dense stromal vascularization, and several ≤ 1 mm regions of positive fluorescein uptake overlying the abscess (Figure 1A). Aqueous flare (grade 2/4) was noted; although, deeper structures (lens, vitreous, fundus) were not visualized due to miosis and severity of anterior segment disease.

The main etiologic differentials for the corneal abscess were bacteria or fungi. Corneal cytology and culture were obtained from an area denuded of epithelium. Cytology, evaluated by a board-certified clinical pathologist, showed neutrophilic inflammation without any evidence of microorganisms. Surgical management was discussed but initially declined. The alpaca was hospitalized and subpalpebral lavage was placed in the lower conjunctival fornix. Intensive medical therapy was initiated while awaiting bacterial and fungal culture results: 0.2 mL voriconazole 1% (compounded into sterile water) q4h, 0.2 mL ofloxacin 0.3% ophthalmic solution (o.s.) q2h, 0.2 mL cefazolin 5.5% (compounded into artificial tears) q2h, 0.2 mL heterologous canine plasma q2h, 0.2 ml atropine 1% o.s. q12h, and 0.2 mL sodium chloride 5% o.s. q6h OD. Meloxicam (60 mg PO q72h) was initiated once the alpaca began eating well. On day 2, a satellite lesion adjacent to the stromal abscess had substantially increased in size by roughly 3 mm, the corneal bullae had worsened, and the pupil remained miotic (Figure 1B); therefore, the frequency of voriconazole and atropine was increased to q2h and q8h, respectively. The subpalpebral lavage line was prematurely removed by the patient 48 h after placement, thus medications were continued by squirting solutions onto the right eye with a tuberculin syringe and 25-gauge needle hub.

On day 3, despite aggressive medical management, the two stromal abscesses had coalesced, the corneal bullae had worsened, the overlying cornea appeared malacic, and the epithelial defect had increased in size to nearly cover the entire abscess (Figure 1C). On day 4, corneal cross linking (CXL) was performed while the patient was standing with no sedation, using the accelerated protocol: topical instillation of 0.1% isotonic riboflavin without dextran (Peschke^®^ M, Huenenberg, Switzerland) at one drop every 2 min for 20 min, followed by UV-A irradiation (365 nm; Nitecore CUC Chameleon LED flashlight with UV, Austin, TX, USA) at approximately 45 mW/cm^2^ for 2.5 min; of note, the light intensity was verified with a UVA/B light meter (General Tools & Instruments, Secaucus, NJ, USA) prior to the procedure. A subconjunctival injection of 0.5 mL voriconazole 1% was performed after CXL. The procedure rapidly halted the keratomalacia, but the corneal abscess size and appearance remained unchanged.

On day 5, bacterial culture was reported negative and the corneal malacia was stable, therefore the frequency of cefazolin, ofloxacin, and plasma was reduced to q4h. However, the corneal stromal abscess continued to increase in size another 2 mm by day 7 (Figure 1D). On day 9, fungal culture revealed *Scopulariopsis brevicaulis* growth, an organism reportedly resistant to several antifungal agents (7–9). With positive growth of a fungus known to be more challenging to treat medically, the expected continued intensity of topical medications, and desire to preserve vision, surgical keratoplasty was now elected (day 10).

A pre-operative packed cell volume (30%) and total solids (6.0 g/dL) were within normal limits. The alpaca was administered fentanyl (2.5 mg/kg, IV) and midazolam (0.25 mg/kg, IV) for pre-operative analgesia and sedation then induced with propofol (3 mg/kg, IV) and ketamine (2.5 mg/kg, IV) and maintained with isoflurane in oxygen following orotracheal intubation. An intra-operative blood gas was within normal limits. The alpaca was positioned in left lateral recumbency. The periocular area was aseptically clipped, prepared, and draped routinely. A large Castroviejo eyelid speculum was placed followed by a stay suture in the ventromedial sclera at the limbus with 5-0 silk to gently manipulate the globe to the desired position, negating the need for intraoperative neuro-muscular blockade.

**Figure 2:**
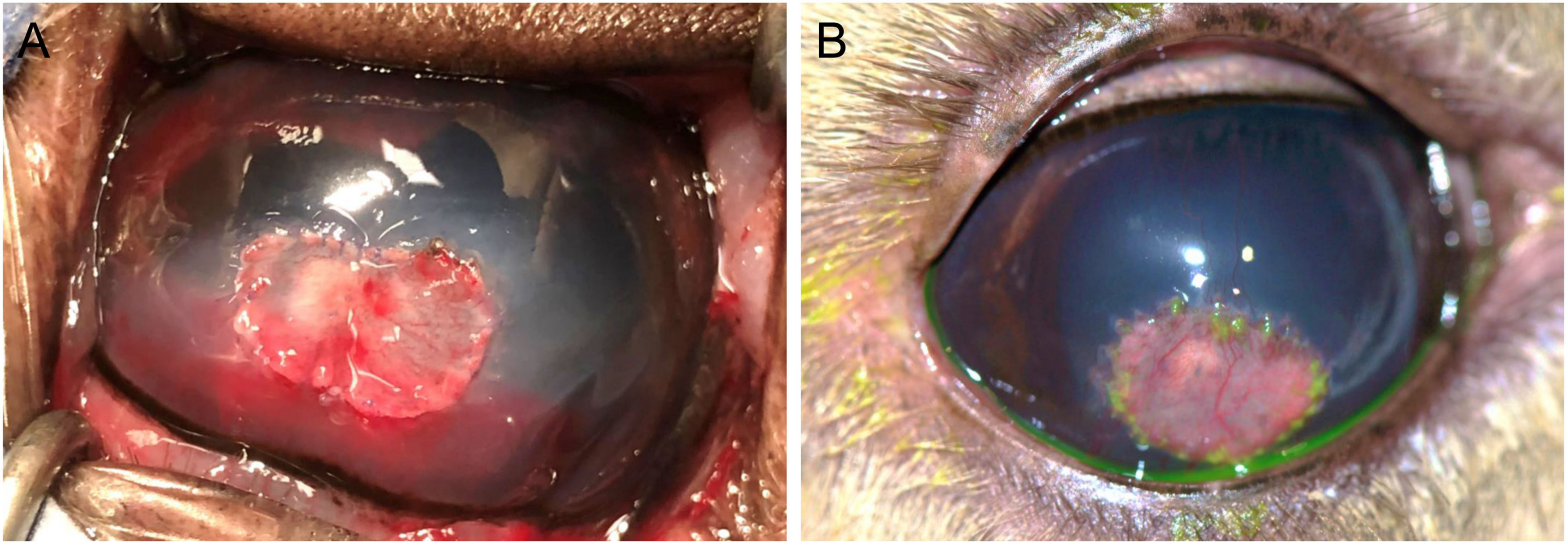
Photographs of the right eye of an 11-year-old female alpaca post-operatively after penetrating keratoplasty for a fungal stromal abscess immediately after surgery **(A)** and 4 weeks after surgery **(B)**. **(A)**–A 3 mm 4 ply porcine small intestinal submucosa graft (Vetrix Plus) was placed within the penetrating keratoplasty site with an 11 × 9 mm island conjunctival graft overlying the lamellar keratectomy site of 50% stromal depth, sutured in place with 8-0 polyglactin 910 suture. **(B)**–The eye was visual and comfortable 4 weeks after surgery with a healthy island conjunctival graft, sutures intact but dissolving, and a dilated pupil.

The corneal abscess measured 10 × 8 mm in size. A #6900 beaver blade was used to incise 0.5 mm away from the diseased cornea to a depth of ~50% then perform lamellar dissection at this depth. A round 3 mm full thickness abscess was unveiled after the lamellar keratectomy was achieved. Graft materials were prepared including an 11 × 9 mm island conjunctival graft harvested from the anterior surface of the third eyelid using a chalazion clamp and Westcott tenotomy scissors and a 3 mm 4 ply porcine small intestinal submucosa graft (BioSIS bioscaffold multilayer, Vetrix Plus, Cumming, GA, USA) obtained with a biopsy punch. Interestingly, the third eyelid proved to have minimal to no connective tissue or cartilage between the anterior and posterior conjunctiva, thus special care was needed during blunt dissection of the conjunctival graft. A penetrating keratoplasty (PK) was performed as previously described in horses (10). In short the following steps were performed: (1) PK was initiated with a 3 mm biopsy punch down to Descemet's membrane, (2) Vetrix Plus was preplaced into the wall of the corneal defect at six o'clock and two additional sutures were preplaced into the Vetrix Plus at three and nine o'clock, (3) a 21-gauge needle was pierced through Descemet's membrane, (4) viscoelastic (Hyvisc, Boehringer Ingelheim, St. Joseph, MO, USA) re-inflated the anterior chamber, (5) corneal section scissors excised the remainder of the corneal abscess, (6) followed by two additional sutures to secure the Vetrix to the PK site. The harvested island conjunctival graft was secured into the lamellar keratectomy site. All corneal and graft sutures were performed with 8-0 polyglactin 910 suture (Figure 2A). A lateral temporary tarsorrhaphy was placed with 5-0 silk suture in an interrupted horizontal mattress pattern. The deep keratectomy sample was submitted for aerobic bacterial and fungal cultures, but histopathology was declined due to financial restraints.

The alpaca's medications were adjusted post-operatively to include: 0.1 mL ofloxacin 0.3% o.s. q4h, 0.1 mL atropine 1% o.s. q8h, 0.1 mL 5% sodium chloride o.s. q8h, 0.1 mL caspofungin 0.5% (compounded into 0.9% sodium chloride) q6h, and ¼ inch strip of terbinafine 1% dermal cream (Lamisil, GlaxoSmithKline, Brentford, UK) OD, as well as meloxicam 60 mg PO q72h. In addition, ceftiofur crystalline-free acid (6.6 mg/kg, SC once) was added for a fever (102.9 °F) of <12 h duration, and pantoprazole (1 mg/kg, IV once daily for 2 days) for suspected third compartment ulceration. Recovery following surgery was otherwise unremarkable. The alpaca was discharged into the care of the owner on day 14 with the following recommendations: 0.1 mL caspofungin 0.5%, 0.1 mL ofloxacin 0.3% o.s., and ¼ inch strip terbinafine 1% dermal cream each q6h, as well as 0.1 mL atropine 1% o.s. q12h OD for 2 weeks then once daily until recheck. The maximum frequency of medications the owners were able to manage at home was q6h. The patient was continued on meloxicam 60 mg PO q72h for 2 weeks.

**Figure 3:**
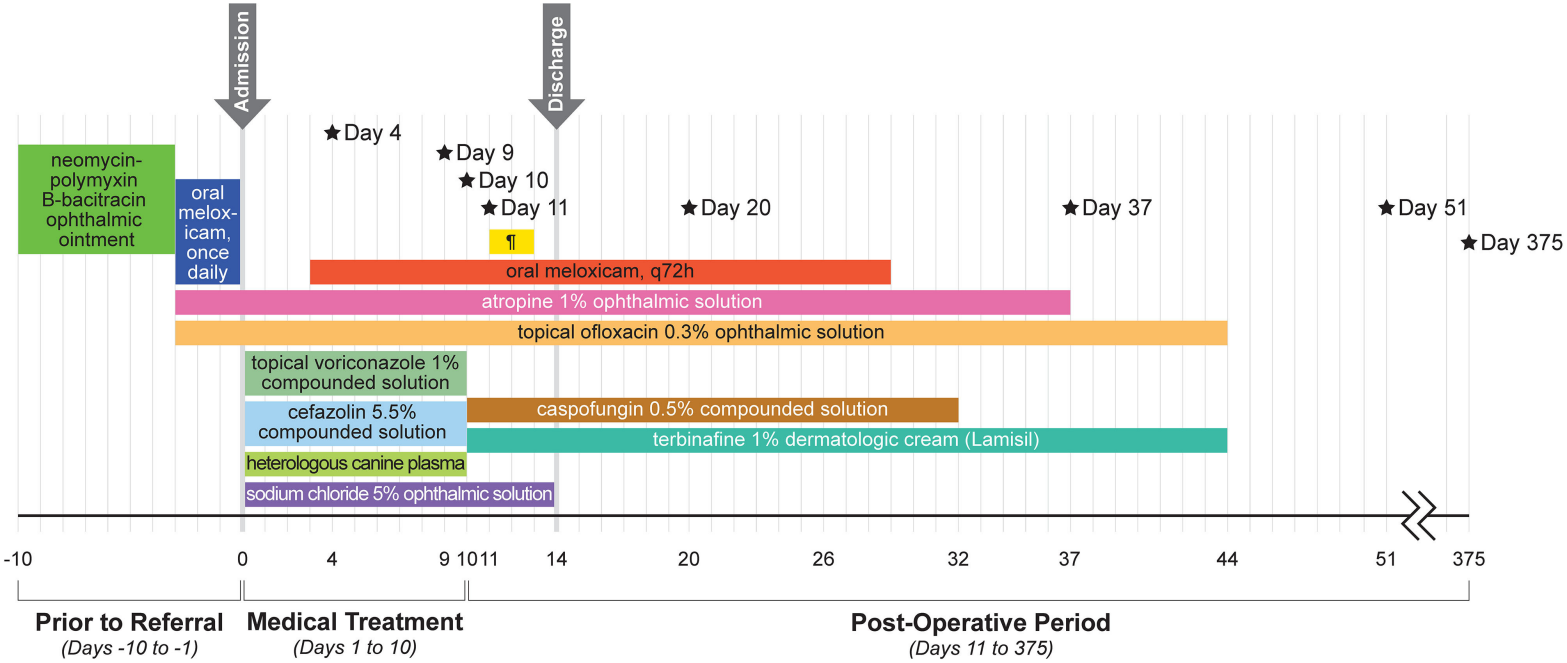
Timeline of the alpaca's clinical course and treatments. Black stars indicate specific treatments and results. Day 4: Corneal cross-linking performed and subconjunctival injection of 0.5 ml voriconazole 1%. Day 9: Culture growth of *Scopulariopsis brevicaulis*. Day 10: Surgical lamellar keratectomy, penetrating keratoplasty, and island conjunctival graft. Day 11: Parenteral ceftiofur crystalline-free acid once. Day 20: Culture growth of *Fusarium verticillioides*. Day 37: Recheck at ISU–considered healed. Day 51: Recheck with rDVM–no relapse off medication. Day 375: Telephone and photo updates with owners and rDVM–small scar, no concerns. Details about the products and dosages can be found in the main text. ¶ = pantoprazole (yellow bar).

Culture results were available on day 20 and reported growth of *Fusarium verticillioides* but no bacterial growth. Due to the higher cost of the caspofungin solution, the owners did not refill this medication and ran out within ~3 weeks (day 32); all other medications were continued as recommended. The alpaca was rechecked 4 weeks after surgery (day 37) and was visual (noted by a positive menace response and behavior observation) and comfortable OD. Intraocular pressures were 16 mmHg OD and 13 mmHg OS as obtained with a rebound tonometer (TonoVet, ICare Finland Oy, Helsinki, Finland) in the equine setting. The dense stromal vascularization had regressed, the island graft was pink and healthy, sutures remained intact but dissolving, and the surrounding corneal edema had resolved (Figure 2B). There was no fluorescein stain uptake. The pupil remained mydriatic and no flare was detected. Small specks of pigment were noted on the anterior lens capsule, but the lens was otherwise clear. Fundic examination was normal. Atropine was discontinued and 0.1 mL ofloxacin 0.3% o.s. and ¼ inch strip terbinafine 1% dermal cream q8h were continued for 1 additional week. The alpaca was rechecked by the rDVM at 6 weeks after surgery and reported to be comfortable and visual with no signs of decline after stopping the medications 1 week prior. Telephone and photo updates with the owner and rDVM 12 months post-surgery confirmed that the alpaca remains visual and comfortable with minimal scarring. A complete treatment timeline is provided (Figure 3).

## Discussion

This study documents the clinical features and therapeutic interventions in an alpaca with a full thickness corneal fungal abscess, adding to the paucity of information about keratomycosis in the species. These two fungal species (*Scopulariopsis brevicaulis* and *Fusarium verticillioides*) have not previously been reported in the cornea of an alpaca and were successfully managed with a combination of surgery (PK) and antifungal therapy (caspofungin 0.5% and terbinafine 1%).

The first fungus *Scopulariopsis brevicaulis* diagnosed in this report was obtained from a superficial swab over the ulcerated cornea. *Scopulariopsis* is considered an uncommon pathogen both within normal commensal flora and clinical infectious keratitis patients. In humans, *Scopulariopsis brevicaulis* is considered a rare but emerging pathogen that is increasingly recognized as a causative agent for superficial and deep mycoses, most commonly in onychomycosis (toenail infections); however, cases of fungal keratitis and endophthalmitis have been reported (7). *Scopulariopsis* was isolated, in conjunction with several bacteria and other fungi, in a single llama with conjunctivitis and non-ulcerated superficial keratitis; however, the significance of that fungus was unknown (1). *Scopulariopsis* was also isolated from the conjunctival or corneal samples in a few healthy horses and horses with ulcerative keratitis (11–14). Moore et al. (11) did not report sensitivity panels for fungi isolated in horses while Betbeze et al. (14) reported that all fungal isolates tested in their study were sensitive to silver sulfadiazine and some were sensitive to natamycin; however, specific minimum inhibitory concentration (MIC) values for the single *Scopulariopsis* isolate were not stated (11, 14).

*Scopulariopsis* sp. have demonstrated resistance to numerous antifungals both *in vitro* and *in vivo* (7–9). One *in vitro* study reported that terbinafine and caspofungin had the best activity of the 11 drugs tested, with low MIC and minimum effective concentrations (MEC) (15). Another *in vitro* study found that triple combination therapy with posaconazole, caspofungin, and terbinafine showed a significant decrease in MIC compared to any agent alone (8). Debridement of infected tissue, in addition to a combination of chemotherapies, has been recommended (7). Successful treatment of corneal *Scopulariopsis brevicaulis* with amphotericin B or natamycin as monotherapies has been reported, although the appropriate therapy is undetermined, as treatment failure has also been reported (8, 9).

The second culture performed from the deep stroma debrided at the time of surgery isolated *Fusarium verticillioides*. *Fusarium* is a common pathogen reported in keratomycoses in horses and humans, and two of the 11 previously reported alpacas with keratomycosis were positive for *Fusarium solani* (3, 6, 10). The therapeutic response of *Fusarium* is variable among its different species (16). *Fusarium* species do not have a consistent MIC and MEC distribution, leading to difficulty in predicting antifungal susceptibility of a single strain (17). Susceptibility patterns in veterinary clinical isolates of *Fusarium* are generally most responsive to polyenes, including amphotericin B and natamycin and are often considered to be multidrug resistant, although only a select set of antifungals were tested in these studies (14, 18, 19). Of note, terbinafine was reportedly the most active drug against 24 clinical isolates of *Fusarium verticillioides* in one study, but terbinafine efficacy was not assessed in the other studies (14, 18–20). Echinocandins (e.g., caspofungin) have variable activity against *Fusarium* species (17, 21).

Medical treatment of keratomycosis should consider susceptibility of the organism to the drug, corneal penetration of the drug, corneal toxicity, or other adverse reactions, and medication cost. Previously reported antifungal medications in camelids with fungal keratitis include silver sulfadiazine, miconazole, itraconazole, and natamycin (1, 3). In this case report, the alpaca was treated with caspofungin and terbinafine, after failure to respond to topical voriconazole, given the reported synergy between these two antifungal medications, especially for *Scopulariopsis brevicaulis*, as well as financial considerations (15).

Terbinafine has variable but often good efficacy against *Scopulariopsis* sp., good efficacy against *Fusarium* sp., is non-irritating to the cornea and very low cost to the client (7, 8, 15, 20, 22–24). Terbinafine is an allylamine antifungal agent and works by inhibiting ergosterol synthesis through inhibition of squalene epoxidate resulting in toxic intracellular accumulation of the precursor squalene (10, 22). Terbinafine is available over the counter as a dermatologic ointment for treatment of fungal skin disease. The dermatologic ointment is not recommended for use in the eye by the manufacturer but was successfully used without any signs of ocular irritation for the treatment of equine keratomycoses and for a rabbit with *Aspergillus fumigatus* keratomycosis, as well as in the case presented here (23). Terbinafine has variable corneal penetration. In rabbits, 0.2% terbinafine suspended in corn oil achieved measurable levels in both the cornea and aqueous humor at a concentration adequate for inhibition of most fungal species (22). However, there was no detectable level of drug in the cornea or aqueous humor after administration of 0.2% terbinafine suspended in castor oil in the horse (24). The variable findings in these reports may highlight a difference in vehicle or species. To the authors' knowledge, no studies have evaluated the corneal penetration of terbinafine 1% dermatologic ointment.

Caspofungin is an echinocandin antifungal agent and works by inhibiting the synthesis of 1,3-d-glucan, leading to cell lysis from increased cell wall permeability (21). Caspofungin is most often reconstituted to 0.5% for eye drops with 0.9% sodium chloride for appropriate osmolality, with stability for 4 weeks under refrigerated conditions (21). Caspofungin penetrates through the cornea with a compromised epithelium (but not intact), resulting in aqueous humor drug levels above MICs for most fungi (21). Caspofungin has variable but sometimes good efficacy against *Scopulariopsis* sp., variable but sometimes good efficacy against *Fusarium* sp., shows no signs of corneal toxicity or irritation, and is intermediate cost to the client (7, 8, 15, 21, 25). No adverse reactions to any topical medications were noted in this case.

In this case report, moderate bullous keratopathy and keratomalacia were noted prior to surgery and improved with CXL treatment. New World camelids have a tendency to develop marked corneal edema following injury, uveitis, and surgery, and is thought to be due to their inherent corneal endothelial pleomorphism and polymegathism (26). CXL is described as treatment for bullous keratopathy in dogs and humans, as well as for keratomalacia in various species, by stopping stromal melting within 24 h and improving corneal edema in the short-term (27, 28). The alpaca in this case report responded favorably to CXL as the progressive keratomalacia and stromal bullae halted after treatment. Since these corneal changes were associated with the infectious keratitis, there was no recurrent corneal edema after healing from the infection. Unfortunately, the evidence to support use of CXL to help eradicate fungal organisms is lacking and inconclusive; (6) which is in agreement with this case's findings.

This alpaca was treated with topical NPB and ofloxacin ophthalmic preparations prior to presentation and diagnosis of keratomycosis. The use of topical antibiotics has been suspected to predispose horses to corneal fungal infections (29). However, a decrease in positive fungal cultures were found after 2 weeks of NPB in normal horses (13). Additionally, in both normal dogs and horses, 1 week of NPB treatment did not significantly disrupt major bacterial taxa as measured by next generation sequencing (30, 31). Conversely, in dogs, topical ofloxacin for 3 weeks after cataract surgery has been shown to lead to a decrease in positive bacterial cultures during treatment, an increase in positive bacterial cultures 3 weeks after stopping treatment, and an alteration in bacterial sensitivity (32). The authors cannot conclude if the topical antibiotic therapy predisposed this patient to keratomycosis, as topical antibiotics are standard of care upon diagnosis of a corneal ulcer and corneal samples were not obtained prior to starting these medications.

The use of a PK has not yet been described in a camelid to the authors' knowledge. However, it is commonly reported in horses to treat full thickness corneal abscesses with a 78% success rate (10). Penetrating keratoplasties are also described for treatment of human keratomycosis, including a case report of *Scopulariopsis* fungal keratitis in a man that failed treatment with amphotericin B and itraconazole, highlighting the importance of debulking the fungal infection when medical therapy fails (33). The authors feel that the large lamellar keratectomy and smaller PK to excise the fungal abscess were paramount in the treatment success. However, it is unknown if this case would have experienced a successful outcome with change in medical therapy alone.

Alpacas are raised for meat in multiple parts of the world (34). However, camelids are not officially considered a food animal in the US and are currently categorized as a minor species (35). Recently, there has been an increase in alpaca meat production in the Northeastern United States, due to interest in the native use of the species as a dual purpose animal, despite lack of regulatory oversight (36). While the alpaca in this case was a companion animal, several aspects of treatment would be impermissible in food animals (e.g., extra-label use of ofloxacin) or would have unknown and presumptive long withdraw recommendations (e.g., antifungals). Thus, the authors recommended the animal to never enter the food chain.

The main limitations of this study were finite in-person follow-up due to geographic restrictions and the lack of susceptibility testing on the specific strains of fungi cultured. Susceptibility testing would have strengthened the speculation that terbinafine and caspofungin were effective in clearing any remaining fungal infection but was not performed due to financial limitations and the prolonged time it takes to obtain fungal susceptibility results.

## Conclusion

The alpaca presented in this report demonstrates that deep keratomycosis can be caused by *Scopulariopsis brevicaulis* and *Fusarium verticillioides*, two previously unreported fungi to cause keratomycosis in camelids. This report also demonstrates successful management with a combination of surgery and topical ophthalmic treatment with caspofungin 0.5% solution and terbinafine 1% dermatologic cream, after failure to respond to voriconazole 1% solution. Combination therapy appears more effective than monotherapy with some fungal organisms, and synergy between antifungal agents is thought to play a role in treatment success. Surgical debulking of the fungal infection is especially helpful in cases that fail initial medical therapy.

## Data Availability Statement

The datasets generated for this article are not readily available because data is limited to the private case records. Requests to access the datasets should be directed to braideefoote@gmail.com.

## Ethics Statement

Ethical review and approval was not required for the animal study because this study was a restrospective case report. Written informed consent was obtained from the owners for the participation of their animals in this study.

## Author Contributions

BF, AB, LS, and RA contributed to the ophthalmic management of the patient. JS contributed to the systemic medical management of the patient. All authors contributed to the manuscript construction, editing manuscript, and review of final submission.

## Conflict of Interest

The authors declare that the research was conducted in the absence of any commercial or financial relationships that could be construed as a potential conflict of interest.
